# Supplementary figures and images for: Phenotypic and Genotypic Characterization of ESBL and AmpC β-Lactamase-Producing E. coli Isolates from Poultry in Northwestern Romania
Source: Antibiotics (Basel). 2025 Jun 5;14(6):578. doi: 10.3390/antibiotics14060578 (PMC12189577; doi:10.3390/antibiotics14060578)

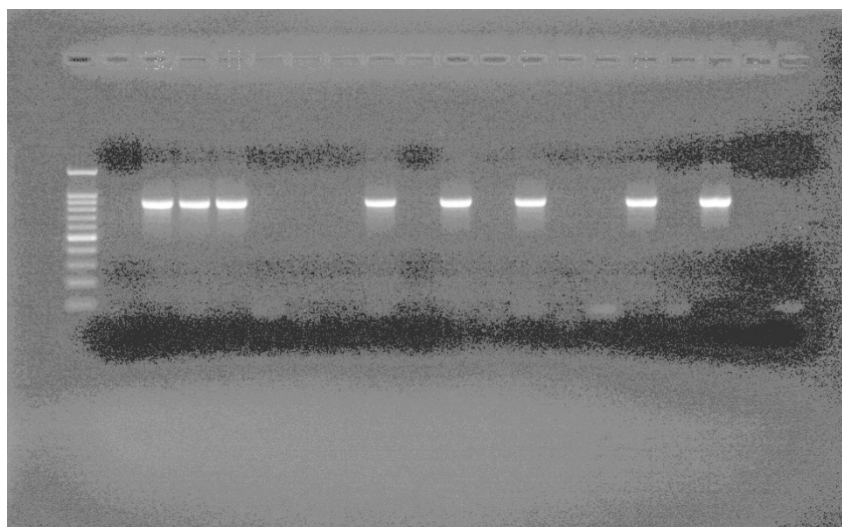

**Figure S1. Agarose gel electrophoresis with resistance gene in *E. coli* (850pB)**

Supplement: Supplementary file 1 [file antibiotics-14-00578-s001.zip › antibiotics-3647186-supplementary.pdf]
